# Supplementary material for: Saccharomyces boulardii (CNCM I-745) improves intestinal damage in sepsis by remodeling intestinal flora structure
Source: Front Cell Infect Microbiol. 2025 Jul 31;15:1602792. doi: 10.3389/fcimb.2025.1602792 (PMC12350329; doi:10.3389/fcimb.2025.1602792)
Supplement: Supplementary file 1 [file DataSheet1.zip › summary/summary/7_Differential_analysis/1_diff_abundance/6_Genus/Group.DW4_vs_CW4_vs_BW4_vs_AW4/Group.DW4_vs_CW4_vs_BW4_vs_AW4_diff_barplot.pdf]

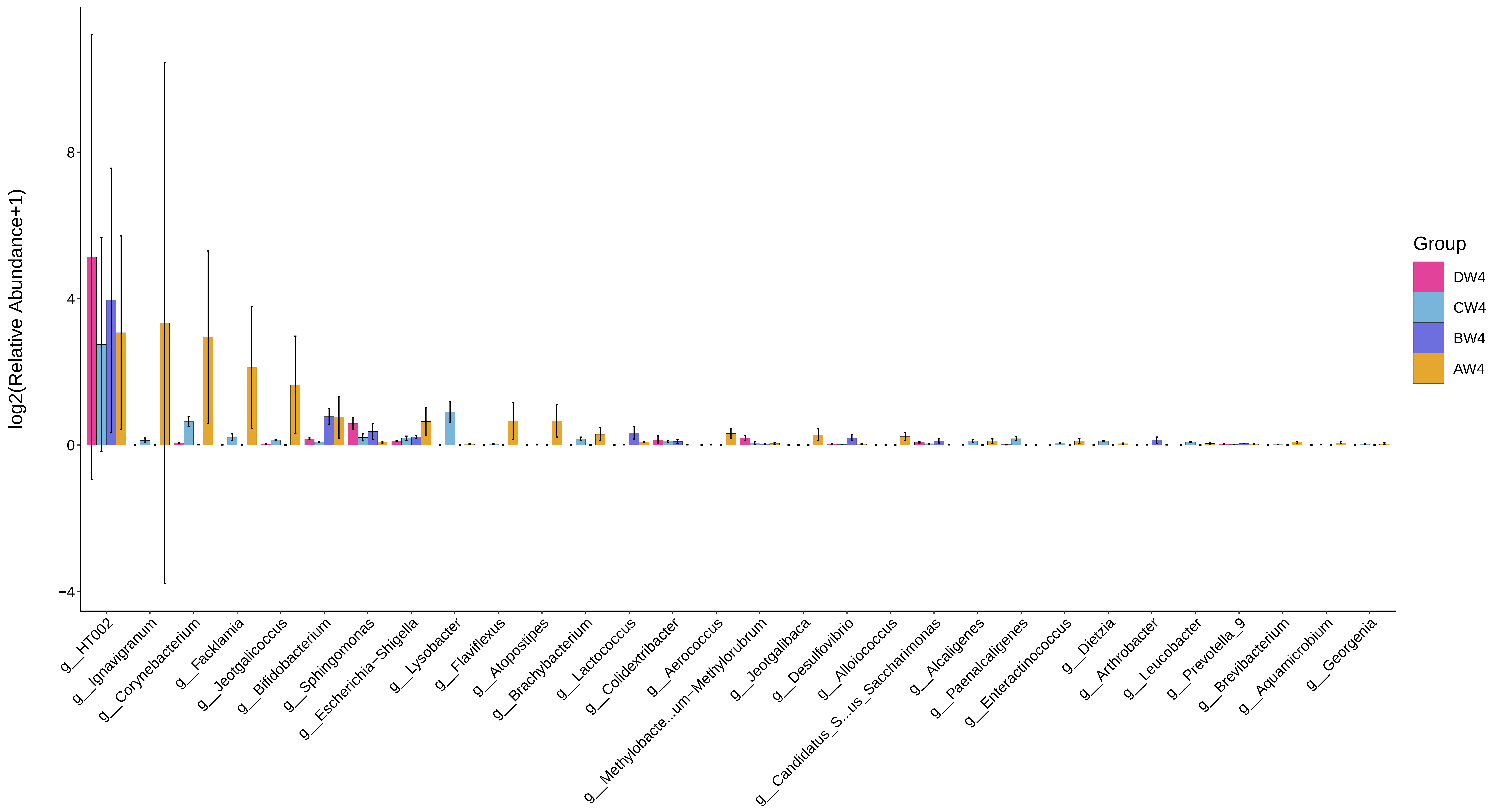

log2(Relative Abundance+1)
8
4
0
−4
g__HT002
g__Ignavigranum
g__Corynebacterium
g__Facklamia
g__Jeotgalicoccus
g__Bifidobacterium
g__Sphingomonas
g__Escherichia−Shigella
g__Lysobacter
g__Flaviflexus
g__Atopostipes
g__Brachybacterium
g__Lactococcus
g__Colidextribacter
g__Aerococcus
g__Methylobacte...um−Methylorubrum
g__Jeotgalibaca
g__Desulfovibrio
g__Alloiococcus
g__Candidatus_S...us_Saccharimonas
g__Alcaligenes
g__Paenalcaligenes
g__Enteractinococcus
g__Dietzia
g__Arthrobacter
g__Leucobacter
g__Prevotella_9
g__Brevibacterium
g__Aquamicrobium
g__Georgenia
Group
DW4
CW4
BW4
AW4
